# Alleviating neuropathic pain mechanical allodynia by increasing Cdh1 in the anterior cingulate cortex

**DOI:** 10.1186/s12990-015-0058-6

**Published:** 2015-09-12

**Authors:** Wei Tan, Wen-Long Yao, Rong Hu, You-You Lv, Li Wan, Chuan-Han Zhang, Chang Zhu

**Affiliations:** Department of Anesthesiology, Tongji Hospital, Tongji Medical College, Huazhong University of Science and Technology, Wuhan, 430030 China

**Keywords:** Anaphase-promoting complex, Cdh1, Synaptic plasticity, Mechanical allodynia, Neuropathic pain, Anterior cingulate cortex

## Abstract

**Background:**

Plastic changes in the anterior cingulate cortex (ACC) are critical in the pathogenesis of pain hypersensitivity caused by injury to peripheral nerves. Cdh1, a co-activator subunit of anaphase-promoting complex/cyclosome (APC/C) regulates synaptic differentiation and transmission. Based on this, we hypothesised that the APC/C–Cdh1 played an important role in long-term plastic changes induced by neuropathic pain in ACC.

**Results:**

We employed spared nerve injury (SNI) model in rat and found Cdh1 protein level in the ACC was down-regulated 3, 7 and 14 days after SNI surgery. We detected increase in c-Fos expression, numerical increase of organelles, swollen myelinated fibre and axon collapse of neuronal cells in the ACC of SNI rat. Additionally, AMPA receptor GluR1 subunit protein level was up-regulated on the membrane through a pathway that involves EphA4 mediated by APC/C–Cdh1, 3 and 7 days after SNI surgery. To confirm the effect of Cdh1 in neuropathic pain, Cdh1-expressing lentivirus was injected into the ACC of SNI rat. Intra-ACC treatment with Cdh1-expressing lentivirus vectors elevated Cdh1 levels, erased synaptic strengthening, as well as alleviating established mechanical allodynia in SNI rats. We also found Cdh1-expressing lentivirus normalised SNI-induced redistribution of AMPA receptor GluR1 subunit in ACC by regulating AMPA receptor trafficking.

**Conclusions:**

These results provide evidence that Cdh1 in ACC synapses may offer a novel therapeutic strategy for treating chronic neuropathic pain.

## Background

Synaptic mechanisms are essential for many neurobiological functions, including learning and memory [1], as well as pathological pain status [2–4]. However, synaptic strength is variable. It is believed that long-term plastic changes, occurring along sensory pathways, from peripheral nociceptors to spinal dorsal horn, and pain-processing brain regions, contribute to nerve injury-induced (neuropathic pain) persistent pain hypersensitivity manifested by spontaneous pain, hyperalgesia and allodynia [2, 3, 5].

Regarding the studies on pain-related synaptic changes, most of the focus in the field is on periphery and spinal dorsal horn, whereas less attention is paid to the long-term cortical plasticity in neuropathic pain conditions. In brain, the anterior cingulate cortex (ACC), an important structure of the limbic system, is believed to be responsible for emotional and attentive responses to the noxious stimuli [6–8]. Cumulative evidence from both human and animal studies demonstrate that, in addition to being involved in affective-motivational pain perception, neurons in the ACC are also important for mediating the sensational component of physiological as well as pathological pain. Stimulation of the ACC facilitate rat nociceptive flexion reflex [9]. Electrical stimulation or local lesion of the ACC can largely reduce acute nociceptive responses, chronic pain in patients, and attenuate mechanical allodynia in rats with neuropathic pain [10, 11].

Synaptic transmission in ACC neurons is significantly increased, and more importantly, pharmacologically blocking this synaptic strengthening, can reduce behavioral hyperalgesia, preventing the development of neuropathic pain [12–14]. Although it has been shown in literature [12–14], that the long-term synaptic changes in ACC are critical for neuropathic pain hypersensitivity, less is known about the molecular mechanisms for this pain-related plasticity. Therefore, understanding the mechanisms responsible for pain-related long-term synaptic strengthening in ACC, and targeting such mechanism will become a novel direction for developing effective neuropathic pain-relieving treatments.

Anaphase-promoting complex/cyclosome (APC/C) and Cdh1, the multisubunit E3 ubiquitin ligase, were an important component of the ubiquitin–proteasome system (UPS). Beyond its roles in cell cycle progression, APC/C–Cdh1 has also been linked to diverse neurobiological functions. Some studies have identified the critical role of APC/C–Cdh1 in regulation of synaptic differentiation and transmission [15]. More recently, Fu et al. reported a mechanism by which APC/C–Cdh1 mediates synaptic plasticity in cortical neurons through an EphA4-dependent signaling pathway [16]. Together, these prompted us to investigate whether APC/C–Cdh1 is involved in long-term plastic changes induced by neuropathic pain, probably acting by ubiquitination and degradation of some presynaptic or postsynaptic component. To address the role of APC/C–Cdh1 in long-term plastic changes induced by neuropathic pain in ACC, we performed morphological, biochemical and behavioral observation with spared nerve injury (SNI) neuropathic pain model in rat, and we also used Cdh1-expressing recombinant lentivirus to validate the molecular mechanism between Cdh1 and neuropathic pain.

## Results

### SNI enhanced c-Fos and GluR1 AMPA receptors expression and changed the synaptic ultrastructure in ACC

**Fig. 1:**
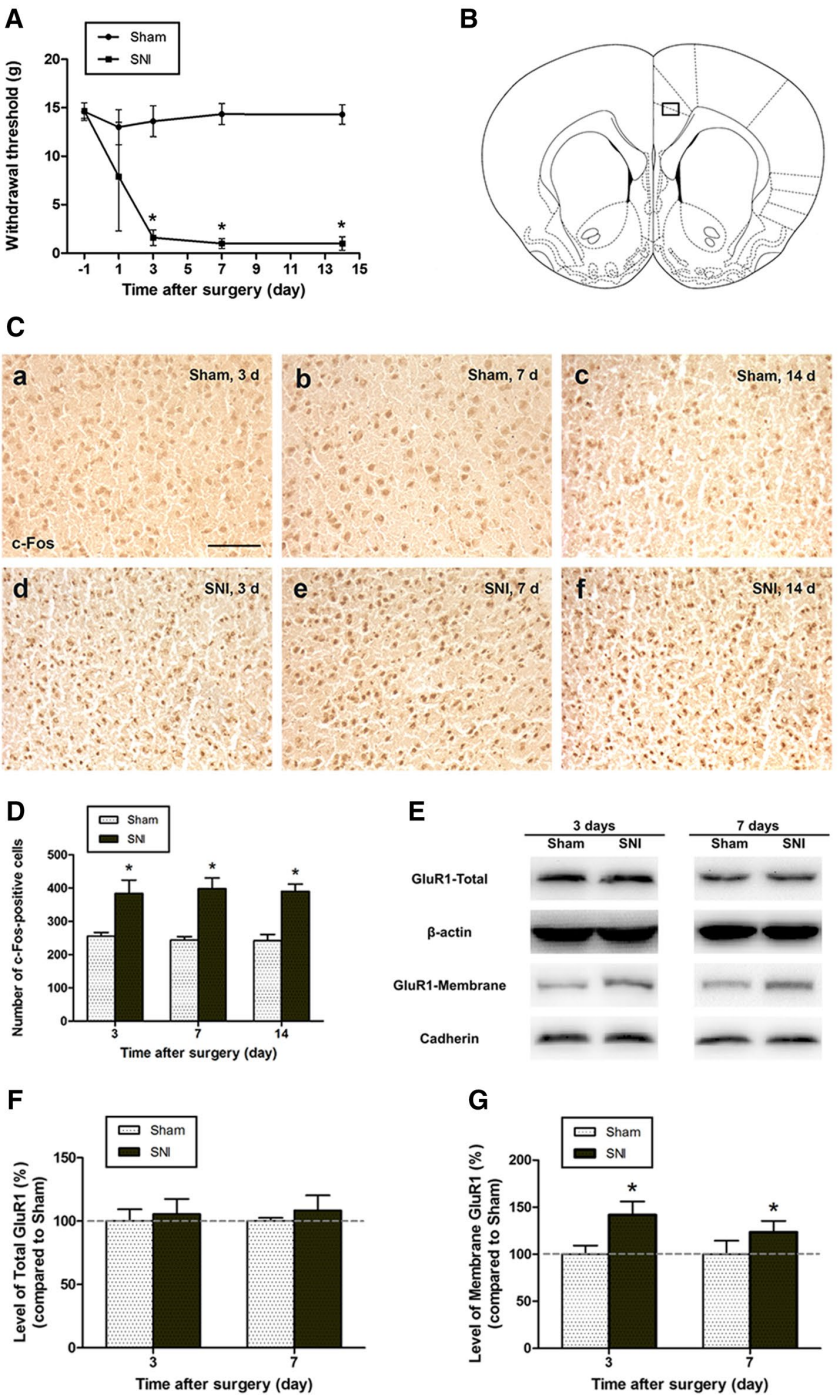
SNI enhanced c-Fos and GluR1 AMPA receptors expression. **A** Mechanical withdrawal thresholds were measured in Sham and SNI rats before and 1, 3, 7, 14 days after surgery. Results are expressed as mean ± SD (n = 6, for each group); *p < 0.05 vs. Sham. **B** Representative rat section through the ACC, 1.7 mm rostral to the bregma. Photomicrographs of the *black box* (0.513 mm × 0.385 mm) used in **C**. C-Fos immunostaining in the ACC of rats 3, 7, and 14 days after nerve injury. *Scale bar* 100 μm. **D** C-Fos-positive cells were counted on both sides of the ACC using the *black box* shown in **B** (n = 3, two sections per rat). Results are expressed as mean ± SD (n = 3); *p < 0.05 vs. Sham. **E** Representative Western blotting showing the redistribution of AMPA receptor GluR1 subunits in the ACC after nerve injury. **F** In the ACC, total GluR1 subunit protein expression was unaffected by nerve injury. Results are expressed as mean ± SD (n = 3, each group); *p* > 0.05 vs. Sham. **G** GluR1 membrane expression in the ACC of Sham- and SNI-operated rats. GluR1 abundance in membrane fractions increased significantly 3 and 7 days after nerve injury (n = 3 per group). *Error bars* SD; *p < 0.05 vs. Sham control

Spared nerve injury produces early, prolonged and robust peripheral neuropathic pain in rats [17]. As previously reported, behavioral mechanical allodynia (i.e. marked hypersensitivity to innocuous mechanical von Frey filament stimulation) was observed in SNI-treated rats, starting on 3 days and persisting at 14 days (SNI vs. Sham, *p* < 0.05; Fig. 1A).

Consistent with the results of behavioral testing, in the ACC, the expression of c-Fos, accepted as a neural marker of pain [18], was significantly increased after nerve injury when compared to the Sham-treated rats. In order to quantify the c-Fos expression in ACC, the c-Fos-positive cell counting was bilaterally performed with two sections per rat (n = 3), approximately 1.7 mm rostral to the bregma, using a microscopic 0.513 mm × 0.385 mm grid under 200× magnification (Fig. 1B). Compared with the Sham group, the number of c-Fos-immunoreactive cells were increased in the SNI-operated rats at 3, 7 and 14 days following nerve injury (*p* < 0.05, Fig. 1C, D).

To further explore the changes in ACC, we also investigated the distribution of AMPA receptor GluR1 subunit in the ACC, 3 and 7 days after SNI. Western blotting showed that induction of neuropathic pain by SNI caused a significant increase in the abundance of GluR1 subunits in membrane fraction (compared with the Sham group, *p* < 0.05) without changing the level of total GluR1 in ACC between Sham controls and rats with SNI procedures (*p* > 0.05, Fig. 1E–G).

**Fig. 2:**
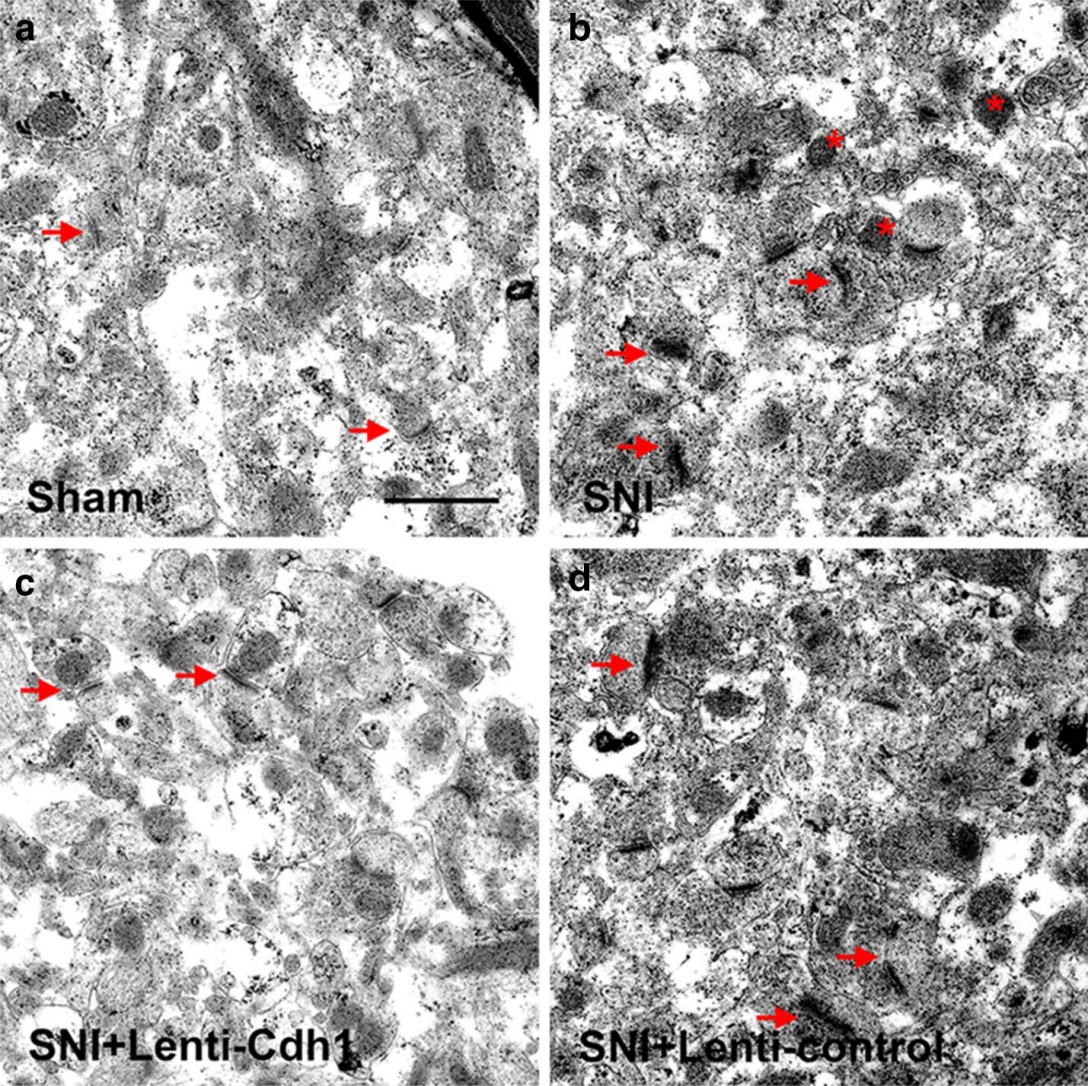
ACC ultrastructure as observed by transmission electron microscopy. **a** In Sham group, Gray’s type I synapses (*arrows*) were identified in the ACC region with regular structures. **b** SNI changed the synaptic ultrastructure in ACC. After SNI, the mitochondria within anterior region of synapse became swollen and increased in number (*asterisks*), the thickness of postsynaptic density was thickened, and the intersynaptic space was decreased, even close to disappearing (*arrows*). The structure of synapses (*arrows*) in SNI + Lenti-Cdh1 group (**c**), but not in SNI + Lenti-control group (**d**), was significantly improved compared with SNI group. Compared with the SNI group, in the ACC, the level of postsynaptic density thickening was reduced significantly, and synaptic cleft was identifiable (**c** *arrows*). *Scale bar* 1 μm

Ultrastructural changes in ACC neuron synapses after nerve injury were examined using transmission electron microscopy. In Sham group, numerous Gray’s type I, excitatory synapses were identified in the ACC region along with regular structures—that is, clear visions of the pre- and postsynaptic membranes, the latter contained electron-dense substance and the former harbored a number of round synaptic vesicles (Fig. 2a). Using transmission electron microscopy, we detected that morphological changes such as numerical increase of organelles, in part, myelinated fibre swollen and axon collapse, occurred to neuronal cells in the ACC of neuropathic pain (SNI) model of rat. Furthermore, the ultrastructures of synapses in ACC were also involved. After peripheral nerve injury, the mitochondria within anterior region of synapse became swollen and increased in number, the thickness of postsynaptic density was thickened, and the intersynaptic space was decreased, close to disappearing (Fig. 2b).

### EphA4–APC^Cdh1^-dependent signaling is involved in SNI-induced redistribution of AMPA receptor GluR1 subunit in ACC

**Fig. 3:**
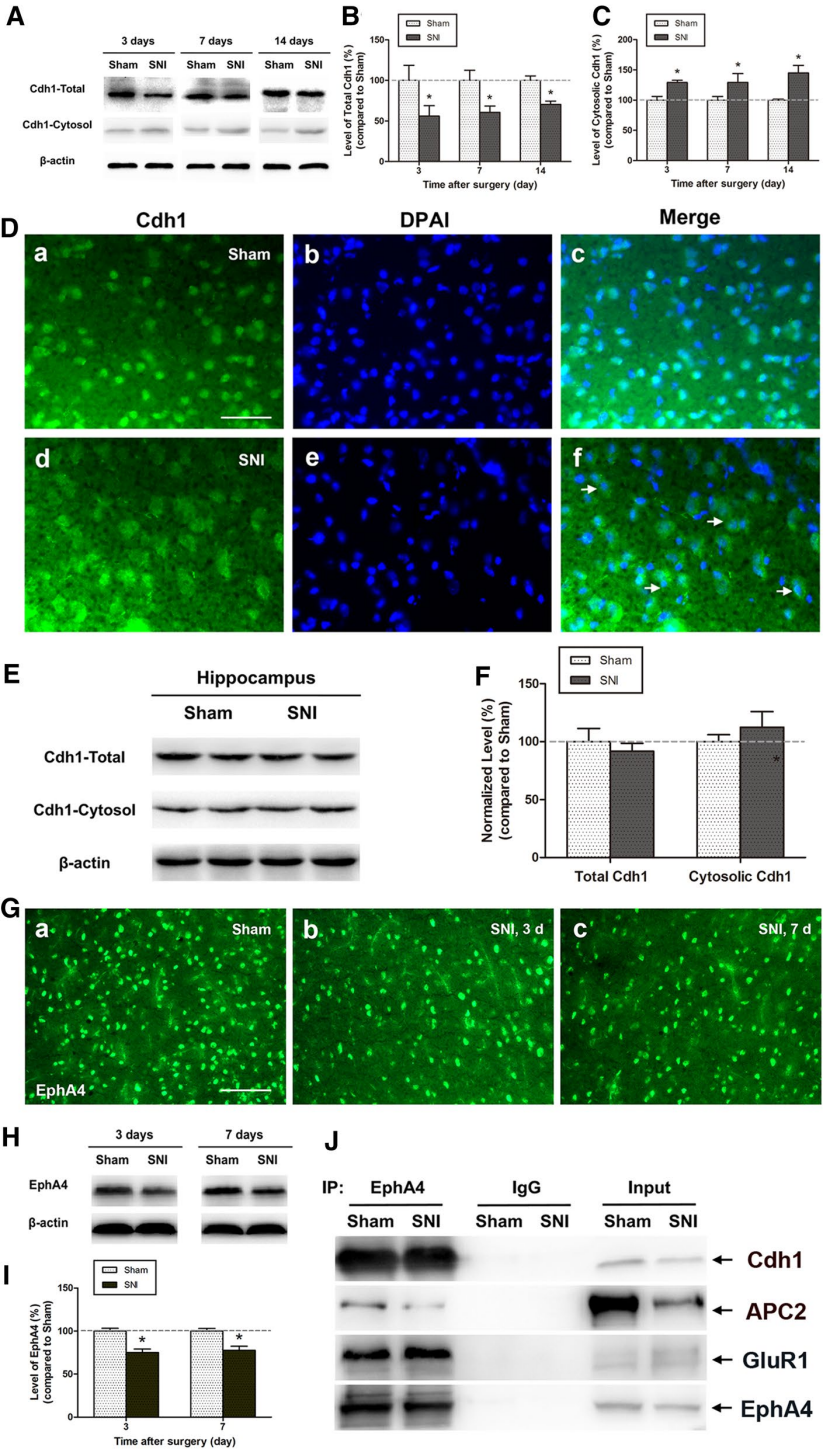
EphA4–APC^Cdh1^-dependent signaling is involved in SNI-induced redistribution of AMPA receptor GluR1 subunit in ACC. **A** Representative Western blotting showing levels of total and cytosolic Cdh1 in the ACC. **B**, **C** Peripheral nerve injury significantly reduced total Cdh1 expression (**B**) and correspondingly increased cytosolic Cdh1 levels (**C**) in the ACC obtained between 3 and 14 days post-SNI (n = 3 per group). *Error bars* SD; *p < 0.05 vs. Sham control. **D** Fluorescence photomicrographs showing the change in Cdh1 intracellular location in the ACC after nerve injury. Sections were labelled with anti-Cdh1 (*green*) and nuclei stained with DAPI (*blue*). Cdh1 was highly expressed in nucleus (*c*), whereas in the SNI group, Cdh1 translocated from the nucleus to cytosol (*f*). *Scale bar* 50 μm. **E**, **F** Total and cytosolic Cdh1 levels in the hippocampus did not change 3 days after nerve injury. Results are expressed as mean ± SD (n = 3, each group); *p* > 0.05 vs. Sham. **G** Immunostaining shows EphA4 expression in the ACC of Sham-operated rats (*a*), and SNI rats at post-operative day 3 (*b*) and 7 (*c*). **H** Representative Western blotting of EphA4 expression in the ACC of Sham and SNI-operated rats. **I** EphA4 levels on day 3 and 7 after nerve injury were significantly lower in SNI-operated rats than in Sham-operated rats. Results are expressed as mean ± SD (n = 3, each group); *p < 0.05 vs. Sham. **J** EphA4 interacted with Cdh1, APC2, and GluR1 in the ACC of Sham- and SNI-operated rats. ACC homogenates (14 days after surgery) were immunoprecipitated with antibodies to EphA4, and immunoblotted with antibodies to Cdh1, APC2, GluR1, or EphA4

In the ACC, the trafficking of GluR1 subunit, as well as AMPA receptor-mediated synaptic transmission is increased markedly during neuropathic pain [14]. To test if EphA4–APC^Cdh1^-dependent signaling is involved in SNI-induced redistribution of AMPA receptor GluR1 subunit in ACC, we examined whether SNI model changed the expression of Cdh1 and EphA4 in ACC cortex. Our experiments, by using Western blotting show that SNI induced a marked decrease of Cdh1 expression in the ACC, as shown in Fig. 3A, B (SNI vs. Sham, *p* < 0.05). Previous studies have shown that as a necessary co-activator of APC/C, phosphorylated Cdh1 translocates from nucleus to cytosol, where it cannot bind to APC/C, resulting in inactivation of APC/C–Cdh1 complex [19]. Based on this, we also conducted experiments to detect possible changes in the intracellular location of Cdh1. In the ACC, the amount of Cdh1 was significantly higher in cytosol 3, 7, and 14 days after SNI, in comparison to the Sham control (*p* < 0.05, Fig. 3A, C). Immunofluorescence staining showed that in Sham group, Cdh1 was consistently highly expressed in nucleus, whereas, after nerve injury, Cdh1 was exported from nucleus to cytosol in ACC (Fig. 3D). To investigate whether the changes in Cdh1 expression and activity were a generalised phenomenon in the brain, we also examined the levels of total and cytosolic Cdh1 in the hippocampus 3 days after SNI. Nerve injury did not change the total and cytosolic Cdh1 levels in the hippocampus (*p* > 0.05, Fig. 3E, F), suggesting that in the brain changes in Cdh1 may be affected in a regionally specific manner. Furthermore, as shown in Fig. 3G–I, the EphA4 expression in the ACC of nerve injury rats was apparently lower than that of Sham-operated controls. To verify our hypothesis, we further examined whether EphA4 interacted with Cdh1, APC2 (a core unit of the APC complex), and GluR1 subunit of AMPA receptor. Indeed, Cdh1, APC2 and GluR1 were co-immunoprecipitated with EphA4 in ACC homogenate. In addition, less EphA4, Cdh1, and APC2 expression, co-immunoprecipitated with GluR1, were observed 14 days after SNI when compared to the Sham-treated rats (Fig. 3J), indicating that a complex of EphA4, GluR1 and APC/C–Cdh1 exists in the ACC and that the interaction between EphA4–APC^Cdh1^ and GluR1 is decreased after peripheral nerve injury. Together, these results provide evidence that EphA4–APC^Cdh1^ complex expression in the ACC of nerve injury rats was lower than control. EphA4–APC^Cdh1^-dependent signaling down regulation was associated with more AMPA receptor GluR1 subunit trafficking to membrane fraction in SNI-induced sensitisation of ACC neurons.

### Intra-ACC microinjection of Cdh1-expressing recombinant lentivirus persistently alleviates the mechanical allodynia induced by SNI

**Fig. 4:**
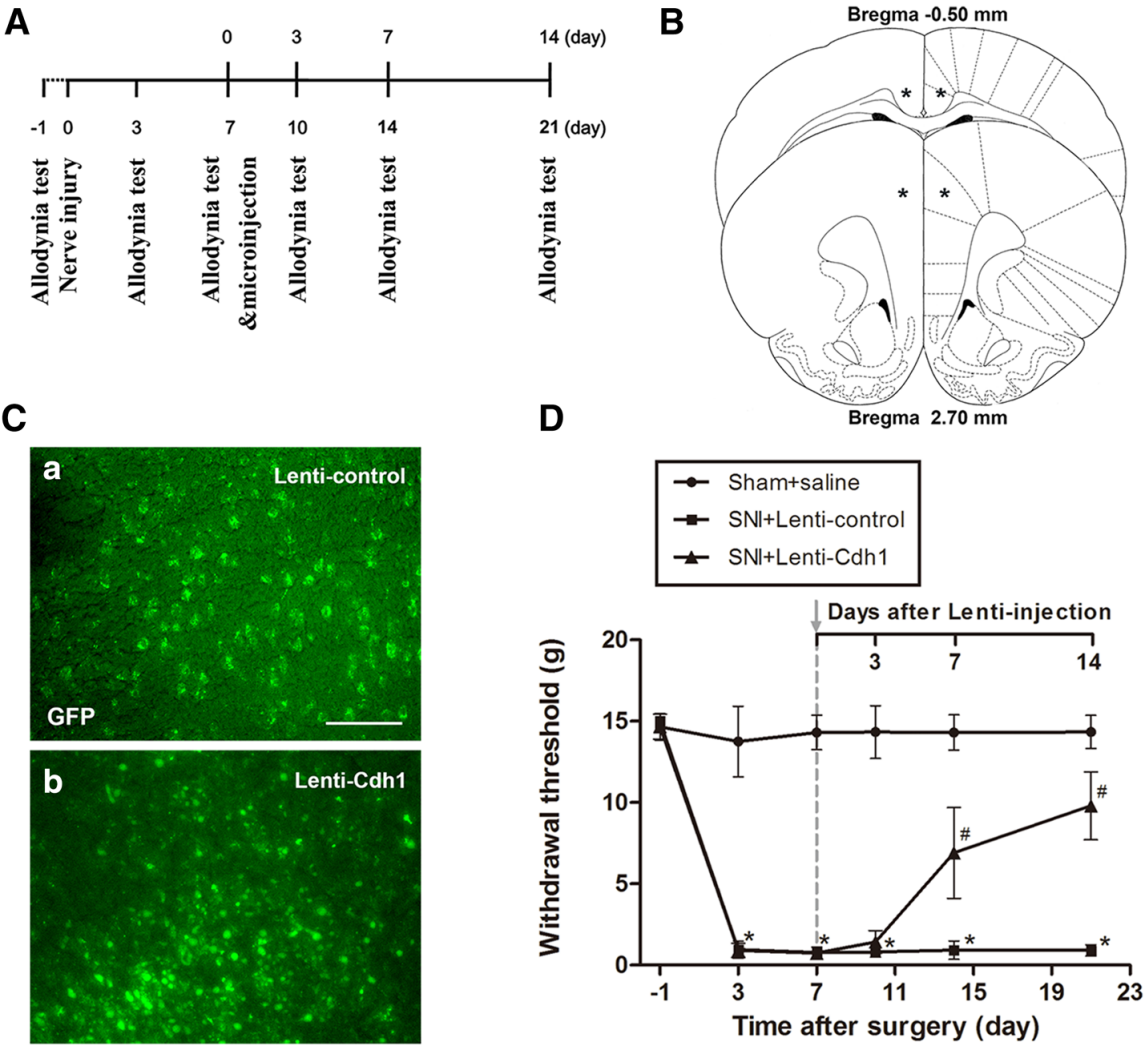
Microinjection of Lenti-Cdh1 into the ACC alleviated established SNI-induced mechanical allodynia in rats. **A** Schematic of the allodynia test and microinjection experiments. **B** Diagram of microinjection location in the ACC. *Asterisks* represented the injection sites. **C** GFP expression was observed 72 h after intra-ACC microinjections of Lenti-control (*a*) or Lenti-Cdh1 (*b*). *Scale bar* 100 μm. **D** Intra-ACC microinjections of Lenti-Cdh1 significantly reduced SNI-induced mechanical allodynia, whereas the Lenti-control injection did not (Sham and SNI + Lenti-control, n = 6; SNI + Lenti-Cdh1, n = 15; *p < 0.05 vs. Sham control; ^#^ *p* < 0.05 vs. Lenti-control)

To investigate the function of down-regulated Cdh1 in the ACC, and whether this down-regulation contributes to the behavioral hypersensitivity after peripheral nerve injury, we carried out intra-ACC microinjections of Cdh1-expressing lentiviral vector (Lenti-Cdh1, 2.0 × 10^8^ TU/ml, 10 μl), which has been previously verified to effectively increase Cdh1 in cultured rat neurons [20], 7 days after SNI (Fig. 4A).

At 72 h after microinjections of Lenti-Cdh1 and Lenti-control, GFP expression was detected in the coronal section through the ACC (approximately 1.7 mm rostral to the bregma) (Fig. 4B) under fluorescence microscope. As shown in Fig. 4C, after Lenti-Cdh1 infection, Cdh1-GFP was observed in nucleus whereas, when infected with Lenti-control, GFP was primarily localised to the cytosol.

Lenti-Cdh1 significantly increased the paw withdrawal threshold (PWT) of the SNI-operated rats: this effect started 7 days after microinjection and maintained for more than 7 days (Lenti-control vs. Lenti-Cdh1 injected group, *p* < 0.05; Fig. 4D). These behavioral data show that microinjection of Lenti-Cdh1 into ACC significantly attenuated the established mechanical allodynia in rats with SNI.

### Intra-ACC microinjection of Cdh1-expressing recombinant lentivirus in SNI rat normalised the GluR1 AMPA receptors expression and the synaptic ultrastructure in ACC

**Fig. 5:**
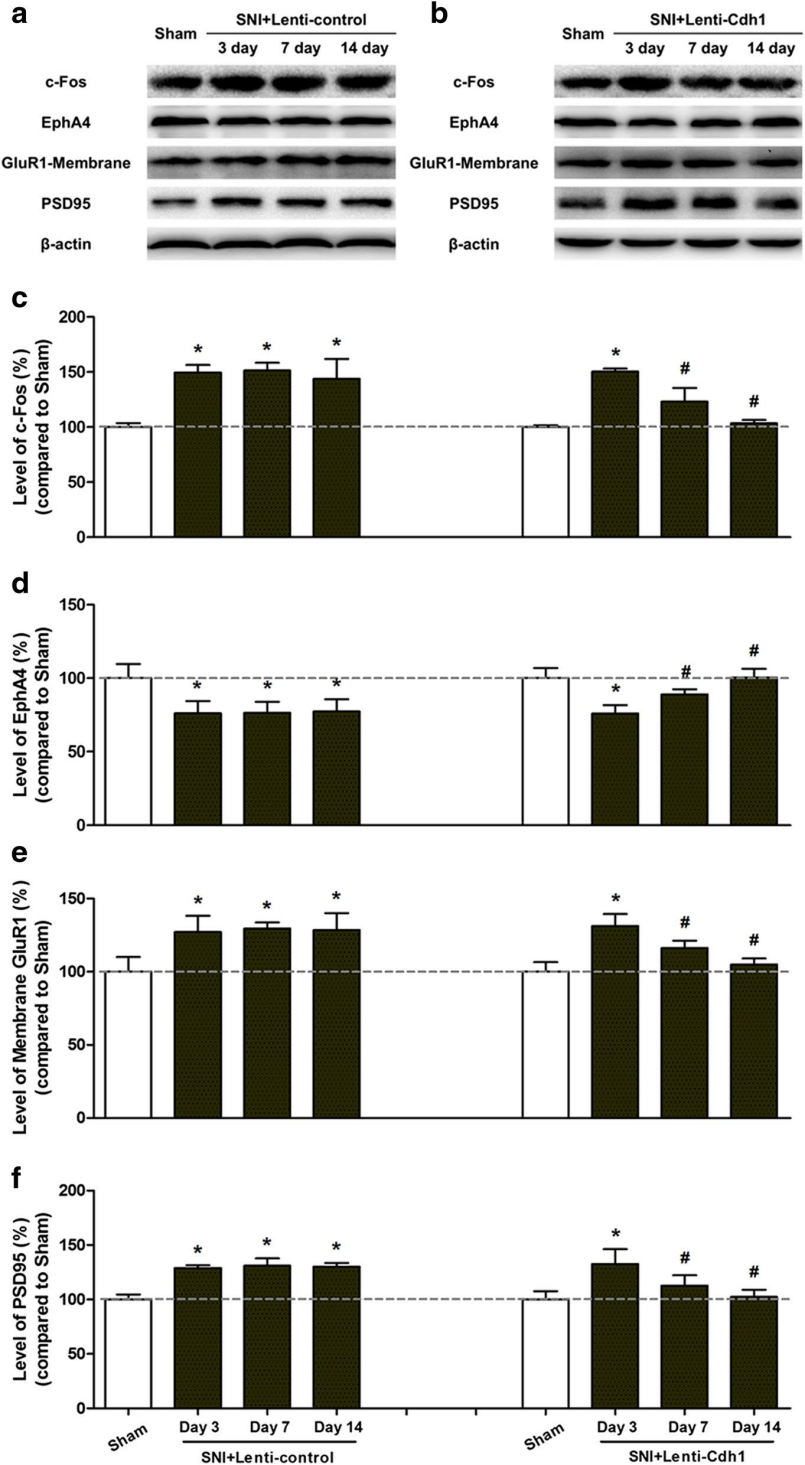
Intra-ACC microinjection of Cdh1-expressing lentivirus normalised SNI-induced redistribution of AMPA receptor GluR1 subunit in ACC. **a**, **b** Representative Western blotting of c-Fos, EphA4, membrane-bound GluR1, and PSD-95 in the ACC of rats subjected to SNI 3, 7, and 14 days following intra-ACC microinjection of control lentivirus (Lenti-control, **a**) or Cdh1-expressing lentivirus (Lenti-Cdh1, **b**). **c** Pooled data showing that Cdh1-expressing lentivirus markedly reduced the SNI-induced increase in c-Fos expression in the ACC 7 and 14 days after lentivirus microinjection (n = 3). *Error bars* SD; *p < 0.05 vs. Sham; ^#^ *p* < 0.05 vs. control lentivirus. **d** Microinjection of Lenti-Cdh1 into the ACC significantly increased EphA4 levels 7 and 14 days after lentivirus injection (n = 3 per group). *Error bars* SD; *p < 0.05 vs. Sham; ^#^ *p* < 0.05 vs. control lentivirus. **e** In the ACC, the SNI-induced increase in cell surface GluR1 expression was gradually returned to control levels 7 and 14 days after microinjection of Lenti-Cdh1. Results are expressed as mean ± SD (n = 3, each group); *p < 0.05 vs. Sham; ^#^ *p* < 0.05 vs. Lenti-control. **f** The SNI-induced increase in PSD-95 protein expression in the ACC was gradually normalised 7 and 14 days after Lenti-Cdh1 injection. Results are expressed as mean ± SD (n = 3); *p < 0.05 vs. Sham; ^#^ *p* < 0.05 vs. Lenti-control

Intra-ACC microinjections of Lenti-Cdh1 or Lenti-control were performed on day 7 following nerve injury. In the ACC of rats with SNI, we found that levels of c-Fos, which is an indicator of increased sensitivity of sensory neurons [18], were significantly decreased 7 and 14 days after Lenti-Cdh1 intra-ACC microinjection (Lenti-control vs. Lenti-Cdh1 microinjection group, *p* < 0.05; Fig. 5a–c). Meanwhile, accompanied with gradual normalisation in the EphA4 level, Cdh1 expressing lentivirus reduced the elevated surface expression of GluR1 protein, which was induced by nerve injury, in the ACC at 7 and 14 days after lentivirus infection (compared with Lenti-control microinjection group, *p* < 0.05; Fig. 5d, e).

As shown in Fig. 2c, d, the structure of synapses in SNI + Lenti-Cdh1 group, but not in SNI + Lenti-control group, was significantly improved compared with SNI group. The form of synapses in the ACC of SNI + Lenti-Cdh1 group was more regular than that in the SNI group, and some synaptic structures were close to the normal. Compared with the SNI group, in the ACC, the level of postsynaptic density thickening was reduced significantly, and synaptic cleft was identifiable, the width of it was apparently increased in SNI rats treated with Cdh1-expressing lentivirus.

### Cdh1-expressing recombinant lentivirus normalised SNI-induced redistribution of AMPA receptor GluR1 subunit in ACC by regulating AMPA receptor trafficking

As the total GluR1 in ACC between controls and SNI rats had no difference, the reduction of surface GluR1 expression after Cdh1 up-regulation may have been caused by ubiquitin specific proteases related to receptor trafficking. To examine this issue, we detected the possible changes in the level of PSD-95, a critical postsynaptic scaffold protein in AMPA receptor trafficking during synaptic plasticity, and its overexpression can reduce the AMPA receptors endocytosis, increase the number of AMPA receptors at synapses [21]. As shown in Fig. 5f, we found, in the ACC, SNI-induced increase of PSD-95 was normalised 7 and 14 days after microinjection of Cdh1 expressing lentivirus (Lenti-control vs. Lenti-Cdh1 microinjection group, *p* < 0.05), suggesting that endocytosis plays an important role for Lenti-Cdh1 caused down-regulation of GluR1.

## Discussion

Neuropathic pain results in plastic changes not only in the dorsal horn of the spinal cord (central sensitisation), but also in supraspinal and cortical areas, including the somatosensory cortices, the prefrontal cortex, the insular cortex, and the ACC [8, 22, 23]. All of these changes contribute to increased pain sensitivity [24, 25]. Here, we focused our study on the ACC, a key cortical area which is not only involved in processing pain-related emotion but also plays a role in the transmission of pain sensation [9–11, 26, 27]. Synaptic activation in ACC induced by nerve injury is critical for the generation and maintenance of neuropathic pain, and more importantly, blocking such pain-related synaptic potentiation, can prevent or alleviate the neuropathic pain hypersensitivity [12–14]. We confirmed SNI induced synaptic ultrastructure change in ACC and found the change was related with EphA4–APC^Cdh1^ regulated redistribution of AMPA receptor GluR1.

Nerve injury-induced c-Fos expression, which is frequently suggested to indicate central sensitisation, persists at least for several weeks in the ACC, presumably resulting from a continued peripheral nociceptive input [18]. The expression of c-Fos has been used to evaluate the neuronal response to a painful experience and to assess the anti-nociceptive effects of many interventions, not only in spinal dorsal horn, but also in the supraspinal structures [18, 28]. Therefore, we also employed c-Fos expression to evaluate the SNI induced change and the effect of Cdh1-expressing recombinant lentivirus in ACC. Some researchers found the up-regulation of c-Fos expression in SNI was asymmetric and it was associated with impaired reversal learning in a right-sided neuropathy [29]. Although we found the SNI induced up-regulation of c-Fos was bilateral in ACC, it will be very interesting to compare if the left-SNI and the right-SNI will lead to a different change in ACC.

Considering the evidence of GluR1 membrane insertion in central sensitisation and pain hypersensitivity [14], we put our focus on AMPA receptor GluR1, to address the molecular mechanism behind the change in ACC. The UPS is one of the major cellular pathways controlling protein turnover in eukaryotic cells. Cdh1 is a co-activator of APC/C, a key E3 ligase that functions as an important component of the UPS. Cyclin-dependent kinase (Cdk)-dependent phosphorylation causes nuclear export of Cdh1, preventing its interaction with the APC/C, thereby limiting APC/C–Cdh1 activity [19]. Our results show a significant decrease and nuclear export of Cdh1 after SNI, indicating that peripheral nerve injury decreases APC/C–Cdh1 activity in the ACC. In recent years, recognition of the role of APC/C–Cdh1 has expanded from its original characterisation as a regulator of cell cycle progression to controlling axon morphogenesis, and in particular, mediating long-lasting synaptic plasticity [16, 30]. APC/C–Cdh1 plays essential roles in synapse development, across model systems, from nematodes and flies to mammals. In drosophila, APC2 loss-of-function mutations lead to increased numbers of presynaptic boutons [31]. At a postsynaptic level, Juo et al. demonstrated that APC/C–Cdh1 regulates GLR-1 recycling, a *C. elegans* non-NMDA class glutamate receptor, to control its abundance at synapses [32]. Recently, endocytosis of the mammalian AMPA receptor GluR1 subunit has also been linked to an APC/C–Cdh1 dependent degradation pathway. In mammalian cortical neurons, APC/C–Cdh1-mediated down-regulation of GluR1 in response to prolonged increase in synaptic activity is thought to be a crucial mechanism for regulating synaptic strength during homeostatic plasticity [16]. In our research, we found APC/C–Cdh1 activity is down-regulated in neuropathic pain in ACC and that this contributes to synaptic activity up-regulation by modulating AMPA GluR1 subunit trafficking through an EphA4 pathway. Moreover, we detected that morphological changes such as myelinated fibre swollen and axon collapse, occurred to neuronal cells in the ACC of neuropathic pain model in rat. This is consistent with the previous results observed in spinal dorsal horn [33], probably resulting from AMPA receptors trafficking induced excitotoxicity. It will be very valuable to validate the result by quantifying the ultrastructural changes for synapses, axons, and mitochondria in ACC with stereological image analysis.

To validate the function of Cdh1 in neuropathic pain in ACC, we intra-ACC microinjected Cdh1-expressing recombinant lentivirus. Even though we targeted neurons specifically, the Cdh1-expressing lentiviral vector mentioned above was non-selective with no specific promoters to target certain populations of neurons, such as excitatory or inhibitory neurons. Recent studies have reported links between Glutamate/GABA balance in ACC and nociceptive responses, with the overarching idea that GABAergic disinhibition may facilitate glutamate-mediated excitatory transmission in the ACC [34, 35]. Not withstanding this limitation, we have found that Cdh1-expressing recombinant lentivirus in SNI rat alleviates the mechanical allodynia and normalised SNI-induced redistribution of AMPA receptor GluR1 subunit and the synaptic ultrastructure in ACC. These findings indicate that interacting with EphA4, Cdh1 contributes to neuropathic pain-related plastic changes in the ACC by modulating the trafficking of AMPA GluR1 subunits, which may be not exclusive but critical for neuropathic allodynia resulting from peripheral nerve injury. A more thorough mechanistic understanding of Cdh1’s function in these processes is required, and in future, it is important to determine in response to nerve injury, whether the changes in Cdh1 are also affected within other populations of neurons, especially GABAergic/inhibitory neurons.

Endocytosis is important for Lenti-Cdh1 caused down-regulation of GluR1. The molecular details of APC/C–Cdh1 mediated AMPA receptor internalisation remain to be investigated. A large body of evidence indicates that AMPA receptor utilises the clathrin-coated-pit machinery for endocytosis, which is initiated with the association of a clathrin adaptor protein AP2 to the intracellular C-termini of AMPA receptor subunits. It is intriguing to note that the AP2 binding domain contains three lysine residues as potential ubiquitination targets. It is possible that ubiquitination at this domain enhances the binding of GluR1 with AP2 so as to facilitate its internalisation [36]. Moreover, the mechanisms for ordered degradation of APC/C substrates remain incompletely understood. As is mentioned above, nuclear localisation of Cdh1 is important for full activity of APC/C–Cdh1 [19]. However, several Cdh1 substrates reside both inside and outside the nucleus [37], or are even found exclusively outside the nucleus [38]. While so far, it has not rigorously been tested whether extra-nuclear substrates require nuclear import for their Cdh1-dependent degradation or whether APC/C–Cdh1, even though concentrated in the nucleus, may be active outside the nucleus as well.

In the present study, by examining the levels of Cdh1 in hippocampus, we showed that changes in Cdh1 are not a generalised phenomenon in central nervous system. However, our preliminary study reveals an SNI-induced decrease in Cdh1 expression in spinal cord (unpublished data). Results from different studies, including electrophysiology and animal behaviour have demonstrated that ACC activation-induced long-lasting facilitation of spinal nociception might be related to persistent hyperalgesia. More recently, works based on animal models of chronic pain have begun to reveal the possible pathways behind modulation of spinal nociceptive transmission from ACC. Chen et al. identified that pyramidal cells in the ACC send direct descending projecting terminals to the dorsal horn of the spinal cord [39]. Consistent with our findings, AMPA receptor trafficking contributes to the potentiated synaptic transmission of ACC neurons. Recruitment of GluR1 mediated the peripheral nerve injury induced long-term enhancement, especially on these corticospinal projecting neurons of the ACC. Direct descending projecting neurons provides possible pathway for ACC to directly regulate the spinal sensory transmission, and most likely account for our findings that nerve injury decreased the levels of Cdh1 in the ACC and spinal cord. However, ACC also widely connects with relevant regions of the descending modulation system, thus, we cannot rule out the possibility that some of them may also contribute to this process.

Furthermore, our previous study demonstrated that APC/C–Cdh1 inhibits astrocyte proliferation induced by oxygen–glucose deprivation and reperfusion, suggesting a role for APC/C–Cdh1 in astrocyte activation during nervous system injury [40]. Activation of glial cells is emerging as key mechanism underlying chronic pain [41, 42]. Given the importance of supraspinal glial activation in descending facilitation of nociception [42], it will be interesting to explore whether glia in the ACC also contribute to pain hypersensitivity, and, if so, whether APC/C–Cdh1 is involved.

## Conclusions

The present study demonstrates that interacting with EphA4, Cdh1 contributes to neuropathic pain-related plastic changes in the ACC by modulating the trafficking of AMPA GluR1 subunits within this cortical structure. Its role in synaptic plasticity is critical for neuropathic allodynia resulting from peripheral nerve injury. Our present findings provide new insights into the pathogenesis of neuropathic pain and identify Cdh1 in ACC synapses as a potential new target for chronic pain management.

## Methods

### Animals

Adult (200–250 g) male Sprague–Dawley rats supplied by Tongji Medical College Experimental Animal Center were used for all experiments. Rats were housed under controlled laboratory conditions (22–25 °C, 12-h alternate light–dark cycles, food and water ad libitum). All animal procedures were performed in strict accordance with the guidelines of the Committee for Research and Ethical Issues of IASP and under protocols approved by the Animal Care and Use Committee of Tongji Medical College, Huazhong University of Science and Technology, Wuhan, China.

### Induction of neuropathic pain

A model of persistent peripheral neuropathic pain was induced by SNI according to the method described by Decosterd and Woolf [17]. Briefly, the SNI procedure involved lesioning two of three terminal branches of the sciatic nerve (tibial and common peroneal nerves) leaving the remaining sural nerve intact. Under anaesthesia with sodium pentobarbital (40–50 mg/kg, i.p.), the common peroneal and the tibial nerves were tightly ligated with a 5–0 silk suture and sectioned distal to the ligation, removing 2–4 mm of the distal nerve stump. Great care was taken to avoid any contact with or stretching of the intact sural nerve. For Sham-operated rats, the sciatic nerve and its branches were exposed, without lesioning. Rats were used for behavioural, morphological, and/or biochemical studies on post-operative days 3–21.

### Behavioural testing

Rats were habituated to the testing environment daily for at least 2 days before basal measurements. Tests were performed by an observer blinded to the treatment protocol during the day portion of the circadian cycle only (06:00–18:00 h). Animals were placed in Plexiglas boxes with a wire grid floor on an elevated platform and allowed to acclimatise for 30 min prior to examination. Using Dixon’s up-down method [43], mechanical allodynia was assessed based on the responsiveness of the injured ipsilateral (left) hind paw to application of a series of von Frey filaments with logarithmically incrementing stiffness (0.4–15.0 g, Stoelting). Licking, biting, and sharp withdrawal of the hind paw were considered positive responses.

### Cdh1-expressing lentiviral vector construction and microinjection

A recombinant rat Cdh1 lentiviral vector was constructed as described previously, which specifically targets rat neurons and enables significant up-regulation of Cdh1 expression [20, 40]. Briefly, the coding sequence of the rat Cdh1 gene (Gene Bank Accession NM_001108074.1) was artificially synthesised and inserted into a pGC-FU vector, resulting in recombinant pGC-FU-Cdh1, which was then recombined with neuron-specific NSE promoter. Production of the Cdh1-expressing lentiviral vector pGC-NSE-Cdh1-GFP (Lenti-Cdh1) was completed by Shanghai GeneChem. Additionally, the same vector backbone was used to generate a negative control (pGC-NSE-control-GFP; Lenti-control) that expresses GFP but not Cdh1. The final titre of Lenti-Cdh1 and Lenti-control were 2.0 × 10^9^ and 4.0 × 10^9^ TU/ml, respectively.

For microinjection, rats were anesthetised by intraperitoneal injection of sodium pentobarbital (40–50 mg/kg). Once anesthetised, the rat’s head was immobilised in a stereotaxic apparatus with incisor bars and non-penetrating ear bars. Following a midline incision, the scalp was retracted to expose the surface of skull. Four small holes were drilled above the bilateral ACC 2.7 mm anterior, 0.5 mm posterior, and 0.6 mm lateral of the bregma according to stereotaxic coordinates of the rat brain. Microinjection was performed using a microsyringe (10 μl), and Lenti-control, Lenti-Cdh1 (2.0 × 10^8^ TU/ml), or saline was delivered into the ACC 2.5 mm ventral to the surface of the skull (2.5 μl/hole, over 5 min). The microsyringe was left in place for 3 min to help prevent back flow. Skin was sutured and cleaned with povidone–iodine.

### Immunohistochemistry

At the indicated time points, rats were deeply anesthetised with an overdose of sodium pentobarbital and immediately perfused transcardially with 0.1 M phosphate buffered saline (PBS, pH 7.2–7.4) followed by 4 % paraformaldehyde in 0.1 M phosphate buffer (PB). Brains were then removed, post-fixed in the same fixative for 2 h, and transferred to PBS containing 30 % sucrose overnight at 4 °C for cryoprotection. Coronal sections of 20-μm thickness were serially cut on a cryostat and collected. From each rat, sections through the ACC (approximately 1.7 mm rostral to the bregma) were selected and used for c-Fos, Cdh1, or EphA4 immunohistological staining. Sections were first blocked with 5 % bovine serum for 40 min at room temperature, and subsequently incubated overnight at 4 °C with the following primary antibodies: c-Fos (rabbit, 1:250, Abcam), FZR1/CDH1 (rabbit, 1:100, Beijing Aviva), and EphA4 (mouse, 1:50, Santa Cruz). Immunohistochemistry with c-Fos was performed using a standard avidin–biotin–peroxidase complex (ABC) method. Sections were incubated in biotinylated goat anti-rabbit IgG for 60 min and followed by avidin–biotin complex for 30 min at room temperature. After rinsing with PBS (3 × 10 min), sections were incubated with diaminobenzidine (DAB) solution (ABC kit, Vector Laboratories) to visualise immunostained proteins, which were then analysed using light microscopy. For Cdh1 and EphA4, sections were incubated for 90 min at room temperature with Cy2-conjugated goat anti-rabbit or Cy2-conjugated goat anti-mouse (1:200, Jackson ImmunoResearch) antibodies, and 4′,6-diamidino-2-phenylindole (DAPI) was used to stain nuclei. Signals were visualised under a Nikon fluorescence microscope. Control sections were similarly processed, except that the primary antibodies were omitted.

### Western blotting analysis

At various times after SNI induction, rats were anesthetised with i.p. sodium pentobarbital, decapitated, and then the region of bilateral ACCs and hippocampus were dissected. Total protein from ACC and hippocampus tissues were extracted by homogenisation in ice-cold RIPA lysis buffer (Beyotime Biotechnology), supplemented with 0.1 mM phenylmethylsulphonyl fluoride (PMSF) protease inhibitor. Cytoplasmic and membranous proteins were obtained using a nucl-cyto-mem preparation kit (Applygen, China) according to the manufacturer’s instructions. Protein concentrations were determined with a Bio-Rad Protein Assay Kit (Bio-Rad). Samples were heated at 95 °C for 10 min in a loading buffer, and equal amounts of protein were fractionated by sodium dodecyl sulphate-polyacrylamide gel (SDS-PAGE) electrophoresis and then transferred onto polyvinylidene difluoride (PVDF) membranes with a Trans-Blot Cell System (Bio-Rad). After blocking with 5 % non-fat milk in TBST buffer (0.1 % Tween 20, 25 mM Tris, 150 mM NaCl, pH 7.5) for 1 h at room temperature, membranes were incubated overnight at 4 °C with primary antibodies against FZR1/CDH1 (rabbit, 1:500, Beijing Aviva), GluR1 (mouse, 1:100, Santa Cruz), c-Fos (rabbit, 1:500, Abcam), PSD95 (rabbit, 1:800, ABclonal), or EphA4 (rabbit, 1:200, Santa Cruz). As a loading control, blots were probed with antibodies against β-actin or cadherin. Membranes were washed with TBST buffer and further treated with a horseradish peroxidase (HRP)-conjugated secondary antibody for 1.5 h at room temperature. Proteins were then visualised using an enhanced chemiluminescence kit (ECL, Thermo Scientific) and a Chemi-Doc XRS imaging system (Bio-Rad), and quantified using Image-Pro Plus 6.0 (Media Cybernetics).

### Co-immunoprecipitation assay

For co-immunoprecipitation assays, total protein extracts were prepared from bilateral ACCs using immunoprecipitation buffer (Beyotime Biotechnology) containing 0.1 mM PMSF protease inhibitor. After centrifugation at 12,400 rpm for 10 min, 500 μg of protein extract was incubated with 10 μg rabbit polyclonal antibody against EphA4 (Santa Cruz) overnight at 4 °C. The immune complex was precipitated by addition of protein A/G agarose on a rotator at 4 °C for 3 h. Following extensive washes with immunoprecipitation buffer, immunoprecipitates were added to SDS-PAGE loading buffer, heated at 95 °C for 10 min, and then detected by Western blotting analysis.

### Transmission electron microscopy

Anterior cingulate cortex tissue samples (1 mm^3^) were fixed with 2.5 % glutaraldehyde in 0.1 M sodium cacodylate buffer overnight at 4 °C. After fixation, samples were post-fixed in 1 % osmium tetroxide for 2 h, dehydrated through a graded series of acetone and then embedded in Epon 812 medium. Ultra-thin sections of each sample were double-stained with uranyl acetate and observed under a transmission electron microscope.

### Statistical analysis

All data are presented as the mean ± standard deviation (SD). Statistical comparisons were performed with SPSS 17.0 using Student’s *t*-test or a one-way ANOVA followed by Fisher’s least significant difference (LSD) multiple comparison test, as appropriate. The criterion for statistical significance was *p* < 0.05.

## Abbreviations

ACC: anterior cingulate cortex
APC/C: anaphase-promoting complex/cyclosome
UPS: ubiquitin–proteasome system
SNI: spared nerve injury
PBS: phosphate buffered saline
ABC: avidin–biotin–peroxidase complex
DAPI: 4′,6-diamidino-2-phenylindole
PMSF: phenylmethylsulphonyl fluoride
SDS-PAGE: sodium dodecyl sulphate-polyacrylamide gel
